# A preliminary study of post-progressive nail-art effects on in vivo nail plate using optical coherence tomography-based intensity profiling assessment

**DOI:** 10.1038/s41598-020-79497-3

**Published:** 2021-01-12

**Authors:** Sm Abu Saleah, Pilun Kim, Daewoon Seong, Ruchire Eranga Wijesinghe, Mansik Jeon, Jeehyun Kim

**Affiliations:** 1 School of Electronic and Electrical Engineering, College of IT Engineering, Kyungpook National University, 80, Daehak-ro, Buk-gu, Daegu, 41566 South Korea; 2 Production Engineering Research Institute, LG Electronics, 17790, 222 LG-ro Jinwi-myeon, Pyeongtaek-si, Gyeonggi-do South Korea; 3 Department of Materials and Mechanical Technology, Faculty of Technology, University of Sri Jayewardenepura, Pitipana, Homagama, 10200 Sri Lanka

**Keywords:** Applied optics, Lasers, LEDs and light sources, Optical techniques, Other photonics

## Abstract

Nail beautification is a widely applied gender independent practice. Excessive nail beautifications and nail-arts have a direct impact on the nail structure and can cause nail disorders. Therefore, the assessment of post-progressive nail-art effects on the nail is essential to maintain optimal nail health and to avoid any undesirable disorders. In this study, in vivo nails were examined in control stage, with a nail-art stage, and after removing the nail-art stage using a 1310 nm spectral-domain optical coherence tomography (SD-OCT) system. The acquired cross-sectional OCT images were analyzed by a laboratory customized signal processing algorithm to obtain scattered intensity profiling assessments that could reveal the effects of nail beautification on the nail plate. The formation and progression of cracks on the nail plate surface were detected as an effect of nail beautification after 72 h of nail-art removal. Changes in backscattered light intensity and nail plate thickness of control and art-removed nails were quantitatively compared. The results revealed the potential feasibility of the developed OCT-based inspection procedure to diagnose post-progressive nail-art effects on in vivo nail plate, which can be helpful to prevent nail plate damages during art removal through real-time monitoring of the boundary between the nail plate and nail-art. Besides nail-art effects, the developed method can also be used for the investigation of nail plate abnormalities by examining the inconsistency of internal and external nail plate structure, which can be diagnosed with both qualitative and quantitative assessments from a clinical perspective.

## Introduction

The nail is a convex, hard plate made from a tough protective protein that protects the tips of the fingers and toes. The presence of a fingernail offers protection to the nail bed and also gives counter pressure for the pulp, which is crucial for the tactile feel of the fingers. It is useful for being able to grip small objects as a tool for scratching and scraping, and finally, it enriches the beauty of the fingers^1^. Nail beautification is of interest to millions of women worldwide in attempts to make the nails more attractive. Use of nail hardeners, nail enamel remover, sculptured nails, nail cuticle removers, nail extension, artificial nails, and nail-art are some examples of nail beautification practices. Excessive nail beautifications sometimes cause nail disorders. All the abnormalities that affect the nail, including nail plate, nail matrix, nail bed, proximal nail fold, lateral nail folds, hyponychium, and the underlying distal phalanx, are considered nail disorders. The over-practice of nail beautification can result in various side effects, such as thinning nail plate, discoloration, drying, brittleness, distal onycholysis, crack on the nail plate, fungal nail infection (onychomycosis)^2–7^ and finally, these can turn into fatal nail diseases. The exact figure of nail disease cases caused by nail-art has not been studied to date but the effects of several nail beautification/nail-arts on nail have been reported^2–7^**.** The discoloration and morphological change of nail serve as an important diagnostic tool for the diagnosis of different kinds of diseases such as AIDS, Jaundice, Liver trouble, Anemia, Lung disease, diabetes, thyroid disease, melanoma skin cancer, heart problems, congestive heart failure, etc.^8–12^. So it is important to monitor the fingernails for a long period to detect the gradual discoloration and morphological changes of fingernails for the early diagnosis of post-progressive nail-art effects on fingernails as well as various human diseases.

Recently, several techniques have been applied for the assessment of nail disorders. Ultrasound imaging has been used by several research groups to describe, measure, and detect morphological features and changes of the nails in patients with psoriatic and/or psoriatic arthritis^13–17^. But the ultrasound imaging system has low axial and lateral resolutions and it requires the use of a gel as a coupling agent^15,17,18^. Confocal laser scanning microscopy (CLSM) is a non-invasive imaging technique that has been used for the assessment of water loss from the nail^19^ and the diagnosis of nail onychomycosis^20^. However, low penetration depth (400–500 µm), limited scan area (500 × 500 µm^2^), the requirement of trained personnel, use of oil or ultrasound gel onto the surface of the probe before measuring, are the drawbacks of CLSM imaging^19^. Ex-vivo fluorescence confocal microscopy has been used to diagnose non-pigmented nail tumors^21^. The anatomy of the nail apparatus can be analyzed, and small lesions can also be detected through high-resolution magnetic resonance imaging (MRI)^22^. MRI confirms the diagnosis and correct position of the nail lesion to assist in surgical treatment^23^. Nevertheless, the costly MRI imaging system has numerous protocol variations, and also it requires highly trained expert-personnel for imaging and image analysis^24^. Dermoscopy is a non-invasive and in vivo technique that has also been used for the assessment of nail disorders^25^. Due to the lack of 3D resolution, dermoscopy cannot provide depth information of sample^26^. Therefore, a non-invasive and high-resolution oriented imaging system is necessary to overcome the aforementioned limitations of conventional imaging methods and for the assessment of nail disorders.

OCT is a non-invasive and non-ionized optical imaging technique that offers high-resolution, cross-sectional imaging of internal microstructure of materials and biological tissue structures by computing the echo time delay and backscattered light intensity. OCT is also a cost-effective, compact, and real-time imaging modality with a fast scan rate. The image resolution of this system is 1–15 μm that is 10–100 times better than ultrasound^27,28^. The imaging depth of this system is 2–3 mm in transparent tissue and 1–1.5 mm in highly scattering tissue. OCT has been diversely used as a non-invasive imaging tool in medical diagnosis^29–35^, and additionally entomological studies^36,37^, industrial applications^38–40^, and in agriculture^41–43^. OCT has emerged as a powerful and reliable imaging tool for the assessment of nail disorders in recent decades.

The potential utility of comparing normal and diseased nails through high resolution and in vivo OCT imaging has already been demonstrated in previous studies. The capabilities of the OCT technique were demonstrated in detecting subclinical nail involvement in some psoriasis and psoriatic arthritis (PsA)^44–47^, also the sensitivity (44.4) and specificity (95.8%) of OCT were calculated for nail disease^46^. Scalable wide-field vascular imaging in OCT with high penetration depth can distinguish the anatomical layers of the nail plate and nail beds^12^, thus, the abnormalities in the nail bed can also be detected. OCT has also been employed for the assessment of nail deformities^48^, for the real-time visualization of onychomycosis^49^, and for the assessment of nail involvement in acrodermatitis continua of Hallopeau^50^. Additionally, few attempts of the OCT technique were reported for the measurement of nail thickness^51^, for assessing the morphology of applied nail polish^52^, and for laser-assisted trans-nail drug delivery^53^. The abovementioned OCT studies were mostly related to various nail diseases and abnormalities assessment, not related to the post-progressive nail-art effects on the nail plate.

The fundamental scope of this study was to investigate the potential feasibility of the OCT system as a non-invasive imaging technique to examine post-progressive nail-art effects on in vivo nail plate to avoid nail plate damages during the art removal process and to diagnose the nail plate abnormalities in real-time monitoring. The monitored result revealed some cracks and white patches in the sub-surface layers of the nail plate surface after 72 h of removing nail-art. To assess the feasibility, two dimensional (2D) OCT images of ten fingernails from a volunteer were also analyzed through an in-house signal processing algorithm to measure and compare the backscattered light intensity and nail plate thickness of control and art-removed nails to diagnose the nail-art effects on fingernails. The proposed OCT application can be successfully applied to diagnose nail plate deformities as an effective inspection method to examine and monitor fingernails before and after applying the nail-art.

## Materials and method

### SD-OCT system setup

**Figure 1:**
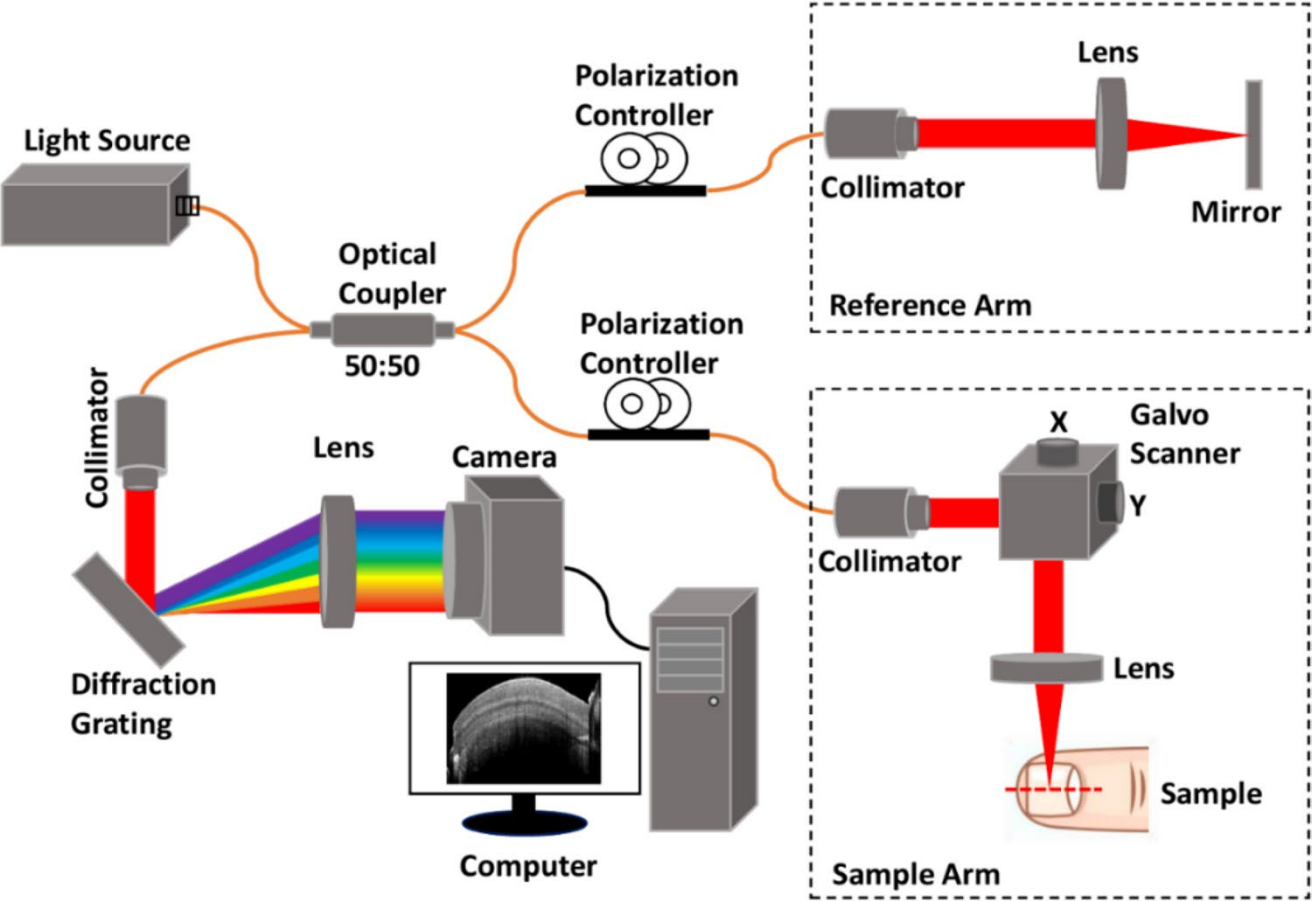
Schematic of the spectral-domain optical coherence tomography system.

A schematic diagram of the SD-OCT system is shown in Fig. 1. The system consists of a broadband light source (EXS210045-01) with 1310 nm center wavelength and full width at half maximum of 100 nm. The output power of this light source is 10 mW. A 50:50 coupler (TW1300R5A2, Thorlabs) was used to split the optical power equally between the sample arm and reference arms. Throughout the entire 15-weeks monitoring period, the optical power of the interferometer was maintained consistently and the sample arm had an average power of 3.4 mW. 2D galvo scanners (GVS002, Thorlabs) were used to scan the samples. The scanning area was 22 mm × 21.5 mm.

For a large scanning area, a 75 mm focusing lens (AC508-075-C, Thorlabs) was used. A 2048-pixel line scan camera (GL2048L-10A-ENC-STD-210) was used in the spectrometer. The measured lateral and axial resolutions of the system were 35 μm and 7.55 μm, respectively, in air. The lateral resolution of the developed system was calculated by using the resolution target (USAF 1951) and the axial resolution was calculated by using the following equation: Axial resolution = (0.44 × *λ*^2^)/Δ*λ*, respectively, Where λ is the center wavelength of the light source and *Δ*λ is the source bandwidth*.* Since the frame rate of the system was 50 frames per second, a single 2D-OCT image was acquired in 20 ms, while a 3D image with 1000 2D-OCT images was acquired within 20 s. The number of pixels of a single 2D-OCT image was 800 × 100 (x × y).

### Sample preparation and imaging process

**Figure 2:**
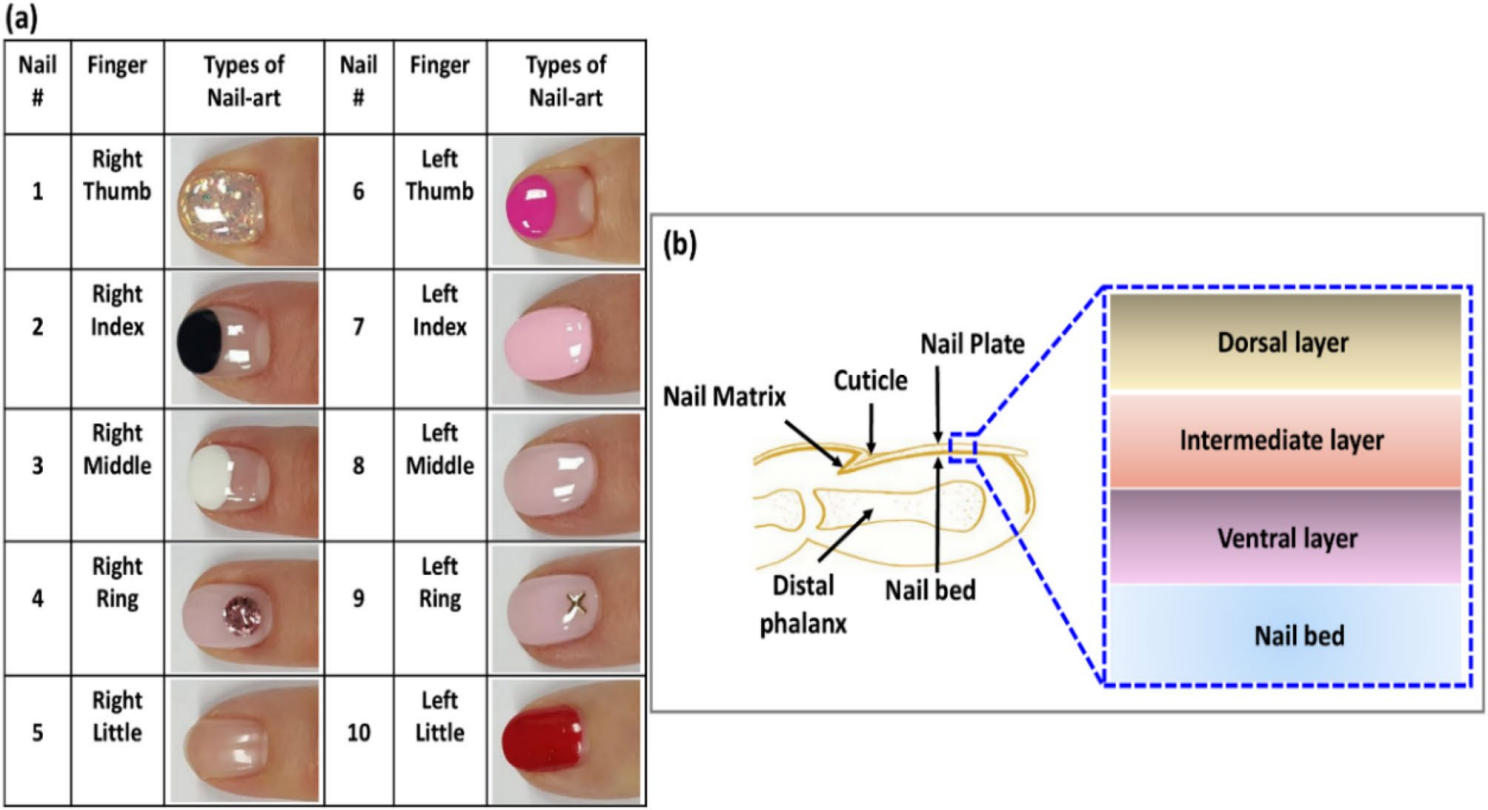
Nail number with corresponding nail-art types and a schematic of finger cross-section with different parts and the nail plate sublayers.

All the human experimental protocols were approved by the Institutional Animal and Human Care and Use Committee of Kyungpook National University (No. KNU-2018-0100), and the methods were carried out in accordance with the approved guidelines and the Declaration of Helsinki. Informed consent was obtained from all subjects. Ten fingernails from a 40-years-old woman volunteer were monitored using OCT imaging for 15 consecutive weeks. Those 15 weeks were divided into three monitoring periods. In the first period (first five weeks), control nails were imaged before the nail-art. After finishing the control nail imaging, nails arts were applied by a nail-art expert. Subsequently, in the second period, OCT imaging was performed on nails with nail-arts. Figure 2a shows the nail numbers and the corresponding nail-art types. After five weeks of nail imaging with nail-arts, nail-arts were removed by the same expert.

The topcoat of nail-art from fingernails 1 to 9 was removed by performing a little grinding by the nail grinder and then washed away the remaining arts by the non-toxic nail polish remover. Red nail polish from the fingernail 10 was removed by using only the non-toxic nail polish remover. To remove the hard topcoat of the nail-art, nail grinding was a mandatory process. where the nail plate safety was maintained carefully. After removing nail-art no special care or treatment was performed on the art-removed nail to obtain the real effect of nail-art on the nail plate. In the third period, OCT imaging was performed on art-removed nails. So in three phases, the total monitoring period was 15 weeks and there was no other reason for taking 15 weeks for imaging. Figure 1 shows the exact orientation and position of the nail sample on the translation stage underneath the sample arm of the imaging system. The schematic of a finger cross-section with different parts and the nail-plate sublayers are emphasized in Fig. 2b.

### Backscattered intensity and thickness measurement algorithm

**Figure 3:**
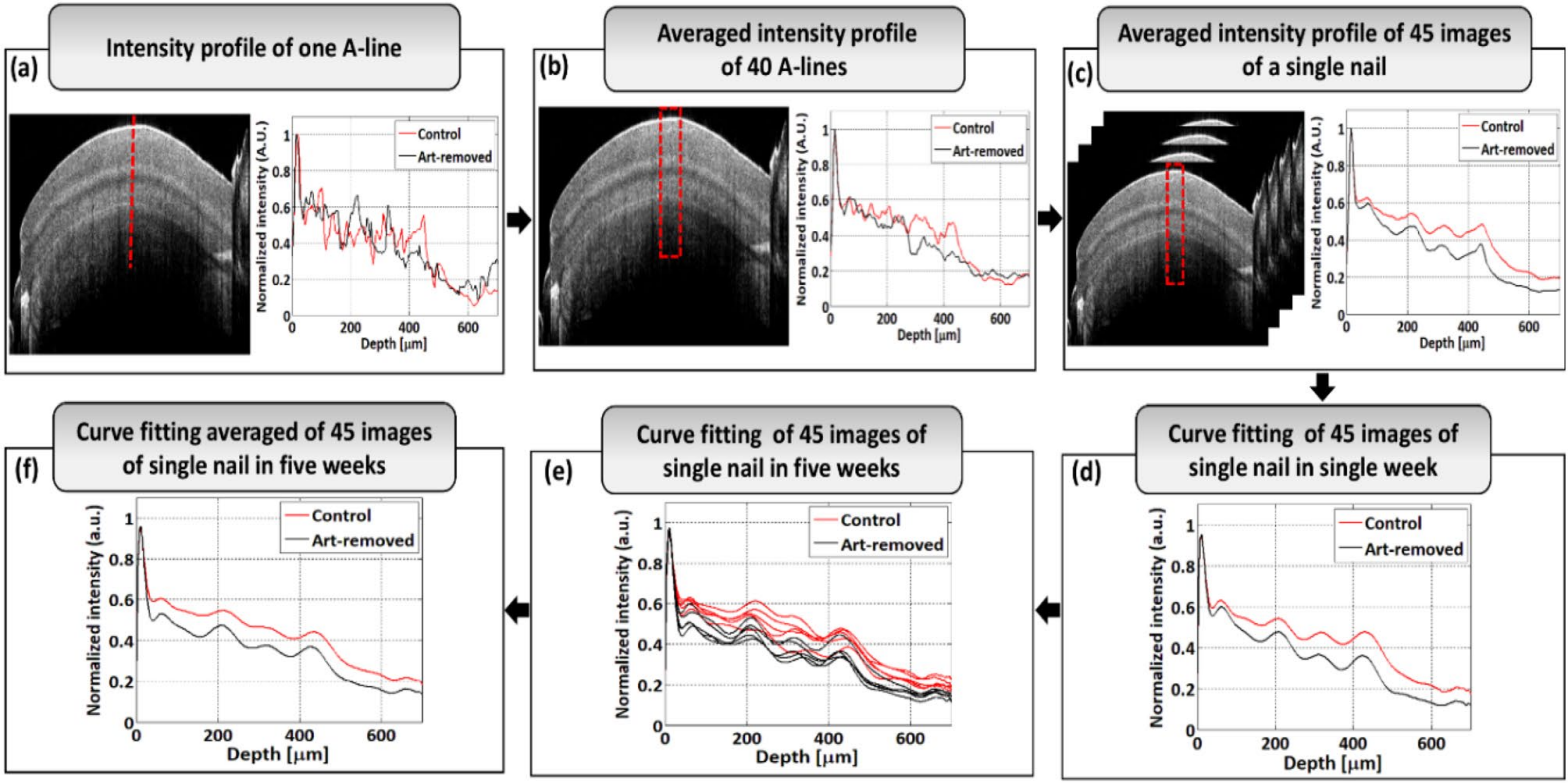
Algorithm of measuring the back-scattered intensity and nail plate thickness. (**a**) Intensity profile of one A-line. (**b**) Averaged intensity profile of 40 A-lines. (**c**) Averaged intensity profile of 45 images of a single nail with 40 A-lines of each image. (**d**) Curve fitting of 45 images of the single nail in a single week. (**e**) Curve fitting of 45 images of a single nail in five weeks. (**f**) Curve fitting averaged of 45 images of a single nail in five weeks.

The depth scan analysis was performed over a specified region of interest (ROI) of nail 2D-OCT images by using a MATLAB coded program to obtain depth intensity profile information. Depth intensity profiles of control and art-removed nail images are shown with red and black colors, respectively. Figure 3a shows the intensity profile of one A-line of a single nail image and the vertical red color dotted line indicates the exact position of depth intensity profile extraction. Figure 3b shows the averaged intensity profile information of 40 A-lines of a single nail image. In Fig. 3b, a region of interest (ROI) is marked with a red color dotted box for the measurement of the back-scattered intensity and nail plate thickness. By using the Matlab program, the intensity profiles of consecutive adjacent 40 columns from the selected ROI of a nail image were taken one by one and then averaged to get the average of 40 A-lines to reduce the noise effect of a single A-line intensity profile of an image. To get a more smooth intensity profile, an averaged of 40 A-lines from each 45 nail images of a nail sample were taken and averaged by using the same process of taking an averaged of 40 A-lines from a nail image. 40 A-lines intensity profiles were taken from the center position of a nail image (shown in Fig. 3b) and then averaged to get a smooth intensity profile. The center position of a nail image was almost flat, thus the post-processing like flattening of the image was not considered. Figure 3c shows the averaged intensity profile of 45 images of a single nail. The acquired intensity value was normalized by dividing the maximum acquired intensity to represent the intensity profile information. The curve fitting algorithm was applied to the averaged intensity profile of 45 images to bypass the unnecessary intensity peaks. Curve fitting, which is usually performed to find a mathematical model that fits the experimental data and to smooth the result, was performed by the “polyfit” and “polyval” MATLAB functions. Figure 3d shows the curve fitting result of 45 images of a single nail sample of a single week. A curve fitting of 45 images of a single nail sample in five weeks was performed to get the backscattered intensity difference between control and art-removed nail images. Figure 3e shows the curve fitting result of 45 images of a single nail sample in five-weeks. Five-weeks curve fitting results of 45 images of a single nail were further averaged to get more comprehensive results in backscattered intensity difference of control and art-removed nail images. Figure 3f shows the averaged curve fitting result of 45 images of a single nail sample in five weeks. A refractive index of 1.47 was maintained for signal processing.

## Result and discussion

### Assessment of nail crack on the nail plate

**Figure 4:**
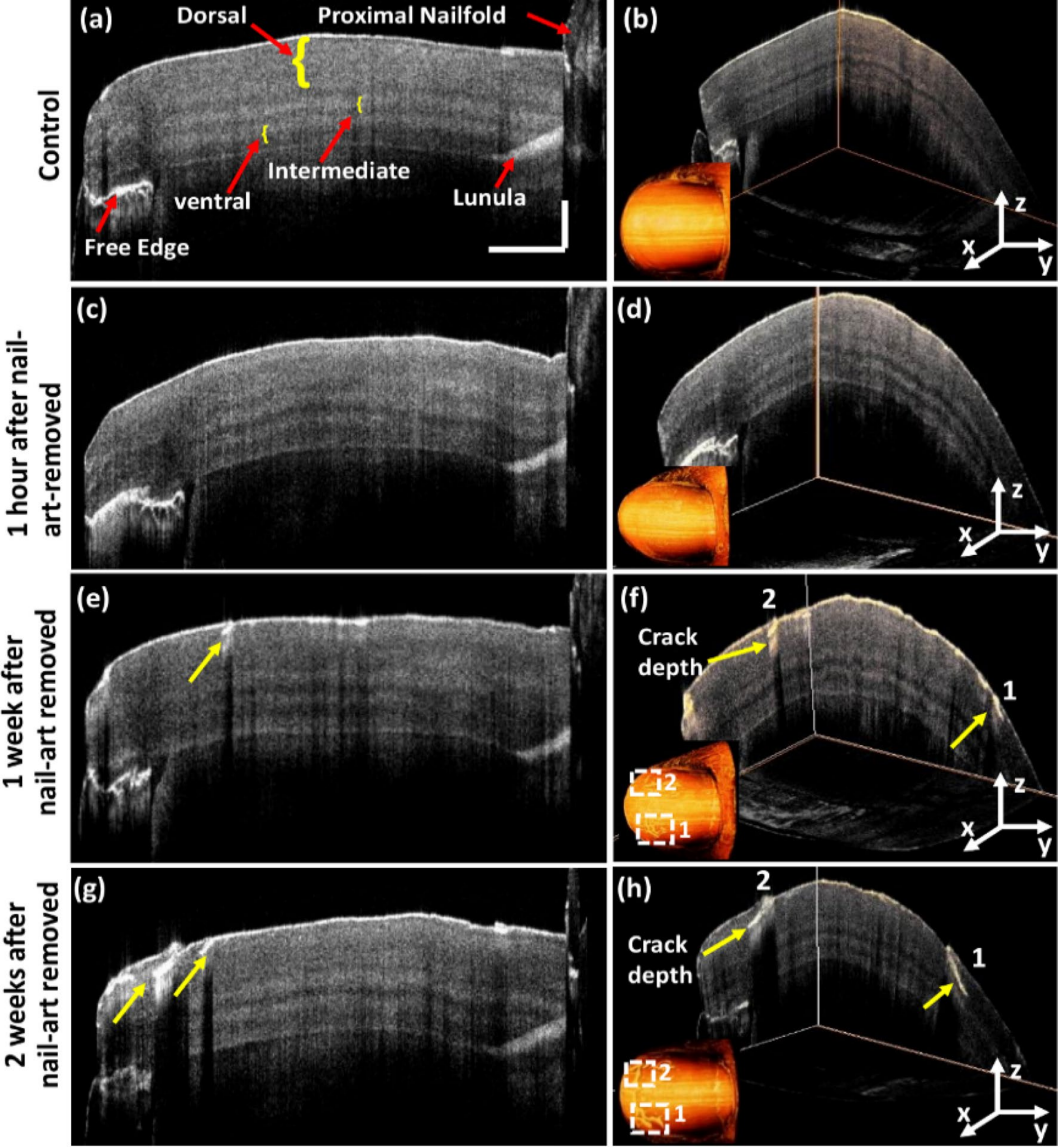
Crack detection on the right-hand thumbnail plate in 2D-OCT and their 3D-enface images at different monitoring periods. Image (**a**) 2D-OCT image of control thumbnail. Image (**b**) 3D-enface of control thumbnail. Image (**c**) 2D-OCT image of right-hand thumbnail after 1 h of nail-art removed. Image (**d**) 3D-enface of the same thumbnail after 1 h of nail-art removed. Image (**e**) 2D-OCT image of right-hand thumbnail after 1 week of nail-art removed. Image (**f**) 3D-enface of the same thumbnail after 1 week of nail-art removed. Image (**g**) 2D-OCT image of right-hand thumbnail after 2 weeks of nail-art removed. Image (**h**) 3D-enface of the same thumbnail after 2 weeks of nail-art removed. The values of horizontal and vertical scale bars are 2 mm and 0.5 mm, respectively.

Figure 4a shows the 2D cross-sectional OCT image of the right-hand control thumbnail. Distinguishable nail plate sublayers and some other parts of the nail, such as proximal nail fold, lunula, and nail-free edge can be identified in Fig. 4a. Nail plate can be split into three sublayers: dorsal, intermediate, and ventral^54,55^ are marked using yellow curly brackets in Fig. 4a. OCT images can visualize lunula because of the increased back-scattering of light from this area. Figure 4b shows the 3D image of the same control thumbnail and its 3D-enface image. The 3D image and the corresponding 3D-enface images of control thumbnail were found without any crack. The 2D-OCT image of the right-hand thumbnail after 1 h of nail-art removal and the 3D-enface of the same thumbnail shown in Fig. 4c,d, respectively. 2D-OCT and 3D-enface images of thumbnail after 1 h of nail-art removal can be identified without any cracks on the nail plate, which has a similar appearance to the control 2D-OCT and 3D-enface OCT images. After 1 week of nail-art removal, both the 2D-OCT and 3D-enface OCT images can be identified with small nail cracks on the nail plate surface (indicated by yellow arrows), shown in Fig. 4e,f respectively. The progression of nail crack on the nail plate surface can be detected in both the 2D-OCT and 3D-enface OCT images after 2 weeks of nail-art removal (indicated by yellow arrows), shown in Fig. 4g,h respectively. The crack positions in 3D and three dimensional enface OCT images are marked by 1 and 2 in Fig. 4f,h.

**Figure 5:**
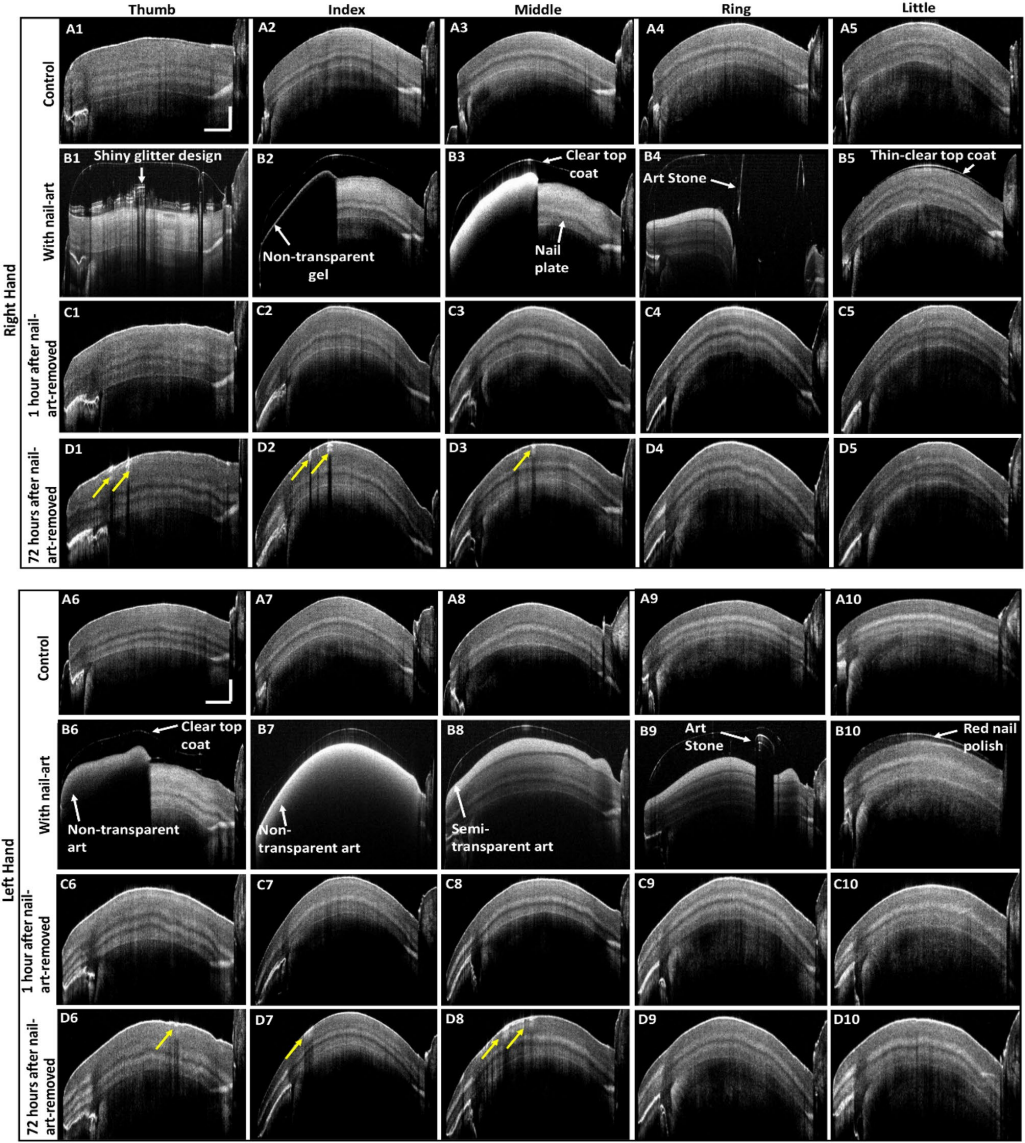
2D OCT images of control, with nail-art, and art-removed nails. Images (**A1**–**A10**) 2D-OCT images of control fingernails of right and left hands. Images (**B1**–**B10**) 2D-OCT images of right and left hands fingernails with nail-art. Images (**C1**–**C10**) 2D-OCT images of right and left hands fingernails after 1 h of nail-art removal. Images (**D1**–**D10**) 2D-OCT images of right and left hands fingernails after 72 h of nail-art removal. The values of horizontal and vertical scale bars are 2 mm and 0.5 mm, respectively.

Control nail images of ten fingernails of right and left hands are shown in Fig. 5A1–A10. Thumbnail control images of both right and left hands with nail plate sublayers are shown in Fig. 5A1,A6, respectively. Other control nails of both hands are visualized with distinguishable nail plate sublayers. Figure 5B1–B10 show the 2D OCT images with nail-art of 10 fingers of both right and left hands. Figure 5B1 shows the 2D-OCT image of right-hand thumbnail with shiny glitter materials and covered with a clear topcoat. A topcoat increases the longevity of the art, and it gives the art a desired look with a smooth finish. Nails 2, 3, and 6 were art with non-transparent gel on the front edge of the nail, and then the whole nail was covered with a clear topcoat as shown in Fig. 5B2,B3,B6, respectively. The front edge of the nail cannot be visible underneath the non-transparent gel. Light can pass through the clear topcoat and visualize the nail plate and other nail-arts underneath the clear topcoat. Figure 5B4 shows that nail 4 was art with a semi-transparent gel covered by the clear topcoat and an additional art stone. Light can partially pass through the semi-transparent gel, and the nail plate can be visualized in Fig. 5B4. However, the nail plate was unable to be visualized underneath the art stone. Nail 5 was art with only a clear topcoat as shown in Fig. 5B5. Nail 7 was fully covered by a non-transparent gel with a clear topcoat, and the nail plate cannot be visualized in Fig. 5B7. Nails 8 and 9 were covered with semi-transparent gel and clear topcoat (Fig. 5B8,B9), respectively. One art stone was additionally used in nail 9. Underneath the semi-transparent nail-art, the nail plate can be partially visualized in Fig. 5B8,B9. Nail 10 was art by red nail polish and underneath the nail polish, the nail plate can be visualized, shown in Fig. 5B10. The 2D-OCT images of right and left hands fingernails with nail-art were found without any crack on the nail plate surface.

2D-OCT images of art removed nails of both right and left hands, were imaged 1 h after the nail-art removal, shown in Figs. 5C1–C10. In 1st hour of nail-art removal, the 2D-OCT images of both right and left hands were found without any cracks on the nail plate surface emphasizing an external appearance to the 2D-OCT images of control nails. In the monitoring period of art-removed nails, both right and left hands nails were imaged after 72 h of nail-art removal. Figure 5D1–D10 show the 2D-OCT images of art removed nails of both right and left hands imaged after 72 h of nail-art removal. After 72 h of nail-art removal, the 2D-OCT images of both right and left hands were found with some nail cracks (marked with yellow arrows) on the nail plate surface, shown in Fig. 5D1–D3, and D6–D8, and the formation of nail crack on the nail plate surface was confirmed. Visually no other significant difference was observed between 2D-OCT images of control and art-removed nails of right and left hand after 1 and 72 h of nail-art removal, except some cracks on the art-removed nail plate surface.

**Figure 6:**
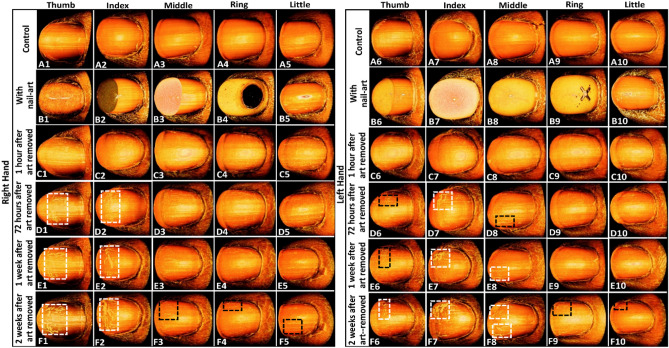
Fingernails 3D images. Images (**A1**–**A10**) 3D images of control fingernails of right and left hands. Images (**B1**–**B10**) 3D images of right and left hands fingernails with nail-art. Images (**C1**-**C10**) 3D images of right and left hands fingernails after 1 h of nail-art removal. Images (**D1**–**D10**) 3D images of right and left hands fingernails after 72 h of nail-art removal. Images (**E1**–**E10**) 3D images of right and left hands fingernails after 1 week of nail-art removal. Images (**F1**–**F10**) 3D images of right and left hands fingernails after 2 weeks of nail-art removal.

Figure 6 shows the fingernails 3D images of control, with nail-art, 1 h after nail-art removal, 72 h after nail-art removal, and 2 weeks after nail-art removal of the right and left hands. The results confirm the clear surface visibility of the control nail plate surfaces without any crack, shown in Fig. 6A1–A10. Figure 6B1–B10 show the top view of fingernails of right and left hands with nail arts. The 3D images of fingernails of the right and left hands were imaged after 1 h of nail-art removal, which can be visualized without any crack, are shown in Fig. 6C1–C10. The 3D images of fingernails of both hands were imaged after 72 h of nail-art removal, are shown in Fig. 6D1–D10. After 72 h, the nail plate surfaces of the art-removed nails can be visualized with some cracks (indicated by white dotted box), shown in Fig. 6D1,D2,D7. The right and left hands fingernails 3D images, which were imaged after 1 and 2 weeks of nail-art removal, are shown in Fig. 6E1–E10 and F1–F10, respectively. The progression of nail cracks on the nail plate surface can be confirmed after 1 week, shown in Fig. 6E1–E2, and E6–E8 with some long-range cracks (indicated by white dotted box). In 2nd week after nail-art removal, the nail cracks can be visualized with more progression compared with 1st week of nail-art removal. Figure 6F1–F2, and F6–F8 shows the more long-range and clear nail crack on the nail plate surface (indicated by white dotted box). Although all fingernails after 1st and 2nd weeks of art-removal are not affected by the long-range crack, they are not as clear as the control nail plate surfaces. Some white patches and surface irregularities are identified in the nail plate surface of 1st and 2nd weeks of art-removed nails (indicated by black dotted box). The 2D cross-sectional and 3D images of control, with nail art, and 1 h after nail-art removal are visualized without any nail crack. After 72 h of removing nail-art, some cracks were detected in the sub-surface layers of the nail plate surface and the progression of cracks was confirmed after 1 and 2 weeks of nail-art removal. The nail-art protects the nail plate in the nail-art period but after removing the nail-art, the nail-art remover makes the nail plate dry and increases the possibility of forming crack on the nail plate.

**Figure 7:**
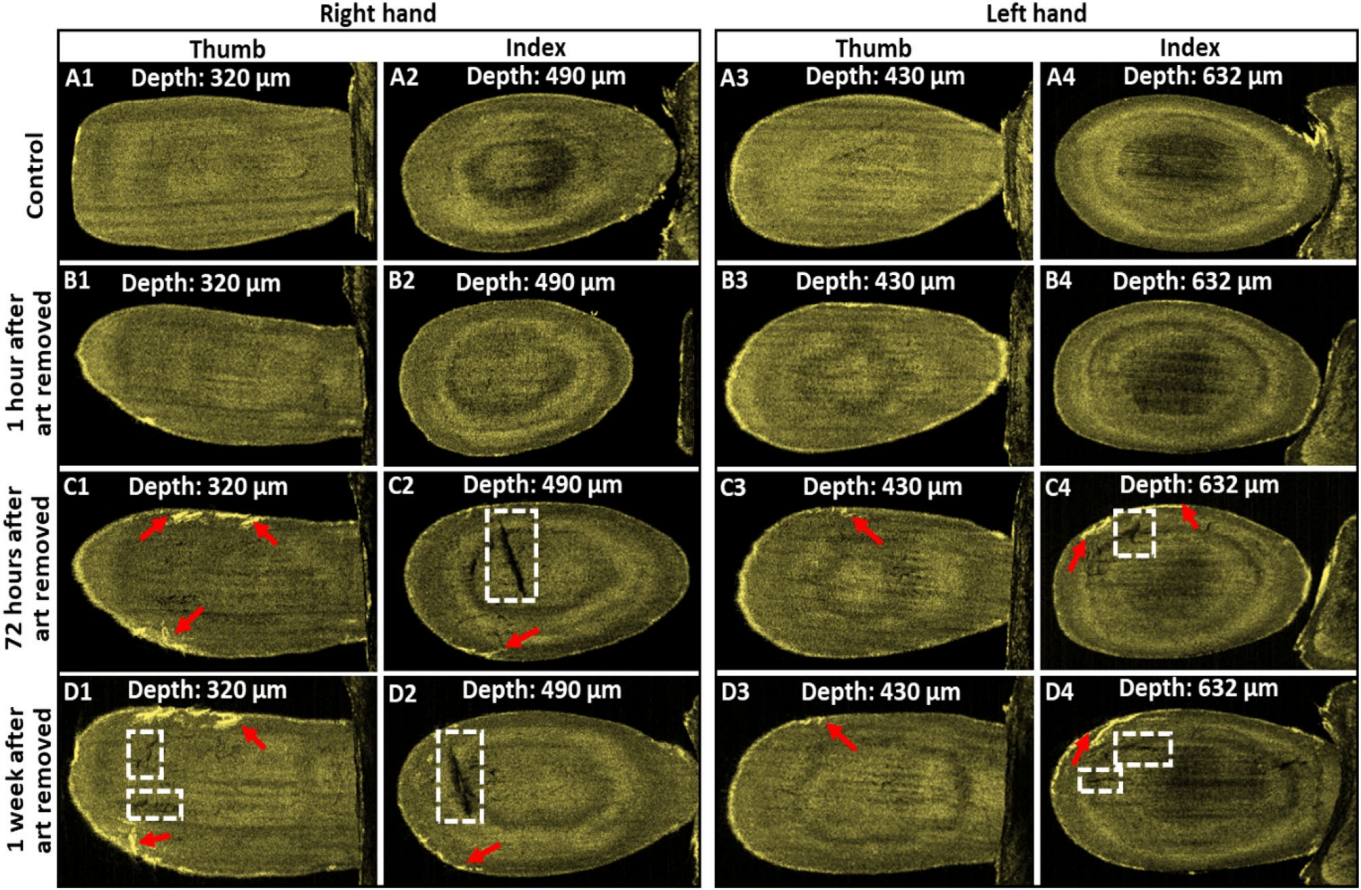
Enface images of control and art-removed nails of right and left hands. Images 7 (**A1**–**A4**) enface images of control thumb, and index fingernails of the right and left hands. Images 7 (**B1**–**B4**) enface images of thumb, and index fingernails of the right and left hands after 1 h of nail-art removal. Images 7 (**C1**–**C4**) enface images of thumb, and index fingernails of the right and left hands after 72 h of nail-art removal. Images 7 (**D1**–**D4**) enface images of thumb, and index fingernails of the right and left hands after 1 week of nail-art removal.

Figure 7 shows the enface images of control and art-removed thumb, and index fingernails of both right and left hands. From the right hand, the control and nail-art removed enface images of thumb and index fingernails were taken at 320 µm and 490 µm depth, respectively. From the left hand, the control and nail-art removed enface images of thumb and index fingernails were taken at 430 µm and 632 µm depth, respectively. Enface images of control thumb and index fingernails of both right and left hands can be visualized without any crack, shown in Fig. 7A1–A4. Also, the enface images of thumb and index fingernails of both hands after 1 h of nail-art removal were found without any crack on the nail plate, shown in Fig. 7B1–B4, which has a similar appearance to the control enface images. But the formation of nail crack can be confirmed (indicated by red arrows) on the nail plate surface of the enface images of thumb and index fingernails of both hands after 72 h of nail-art removal, shown in Fig. 7C1–C4. The progression of nail cracks on the nail plate surface can be confirmed with some long-range cracks after 1 week of nail-art removal, shown in Fig. 7D1–D2. In art-removed enface images of both hands, some black spots (indicated by the white dotted box) can be visualized. The nail cracks on the nail plate surface created a total reflection of scanning laser light during imaging and underneath the nail cracks light could not pass in the depth direction of the nail plate and created the black spots in enface images. In control enface images of both right and left hands, no black spots can be detected, because there was no total reflection of scanning laser light from the crack-free nail plates of control nails.

### Backscattered intensity difference of control and art-removed nails

**Figure 8:**
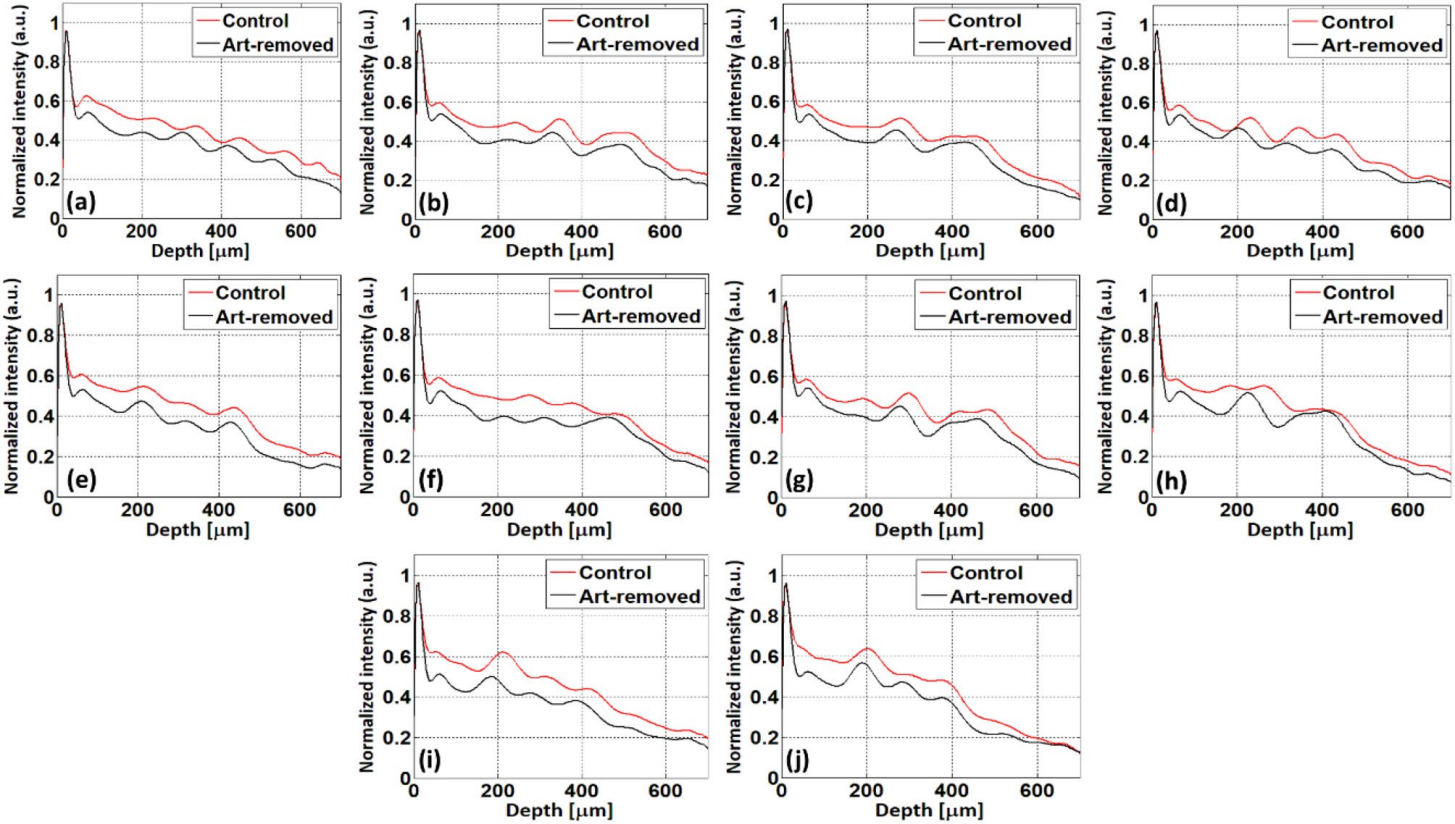
Back-scattered intensity difference of control and art-removed nail images. Images (**a**–**j**) for back-scattered intensity difference of nail 1 to 10.

An intensity difference can be detected between control and art-removed nails. 2D images of control and art-removed nails were further analyzed by a developed signal processing algorithm to get a complete result regarding the backscattered intensity difference. Figure 8 shows the backscattered intensity profiles illustrating the backscattered intensity difference between control and art-removed nails. It can be notified that the backscattered intensity of the control nails is higher than the art-removed nails of all fingers. The varying refractive index and change in the inner biological structure of the nail plate during the long imaging/monitoring period of the art-removed nails are the reasons for degrading the backscattered intensity from the art-removed nails.

### The thickness difference between control and art-removed nails

**Figure 9:**
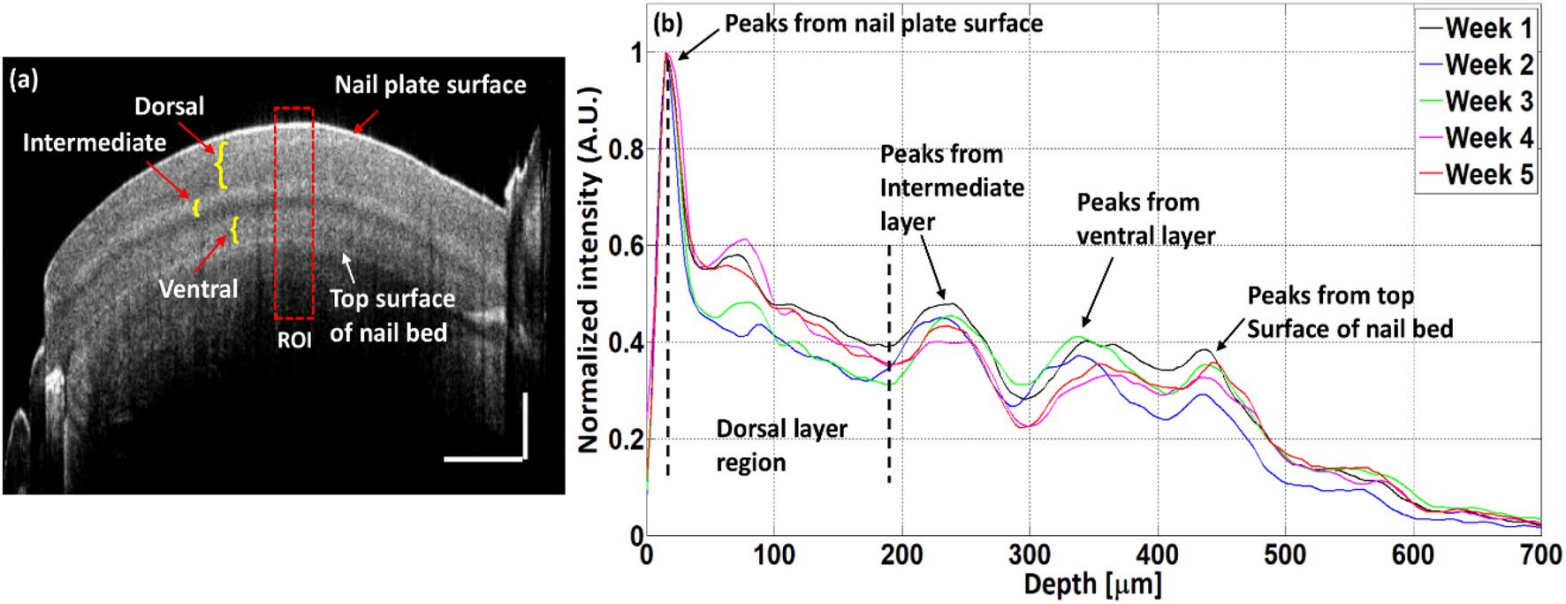
Nail plate thickness measurement process of control and art-removed nails. Image (**a**) 2D cross-sectional OCT image. Image (**b**) depth profiles of a nail in consecutive five weeks of imaging.

The nail-art effect on nail plate thickness was evaluated by an in-house signal processing algorithm based on the intensity profile of the 2D-OCT images. Figure 9 shows the nail thickness measurement process of the control and the art-removed nail. Nail cross-sectional 2D-OCT image and intensity profiles of a nail in consecutive five weeks of imaging are shown in Fig. 9a,b respectively. In Fig. 9a, the nail plate thickness measurement ROI, nail plate sublayers (dorsal, intermediate, and ventral), and nail bed top surface are indicated by the red dotted box, yellow curly brackets, and by the white arrow, respectively. An ROI of 1 × 1 mm area was selected from the center of the nail plate for measuring nail plate thickness of both control and art-removed nails as explained earlier in the backscattered intensity and thickness measurement algorithm section.

**Figure 10:**
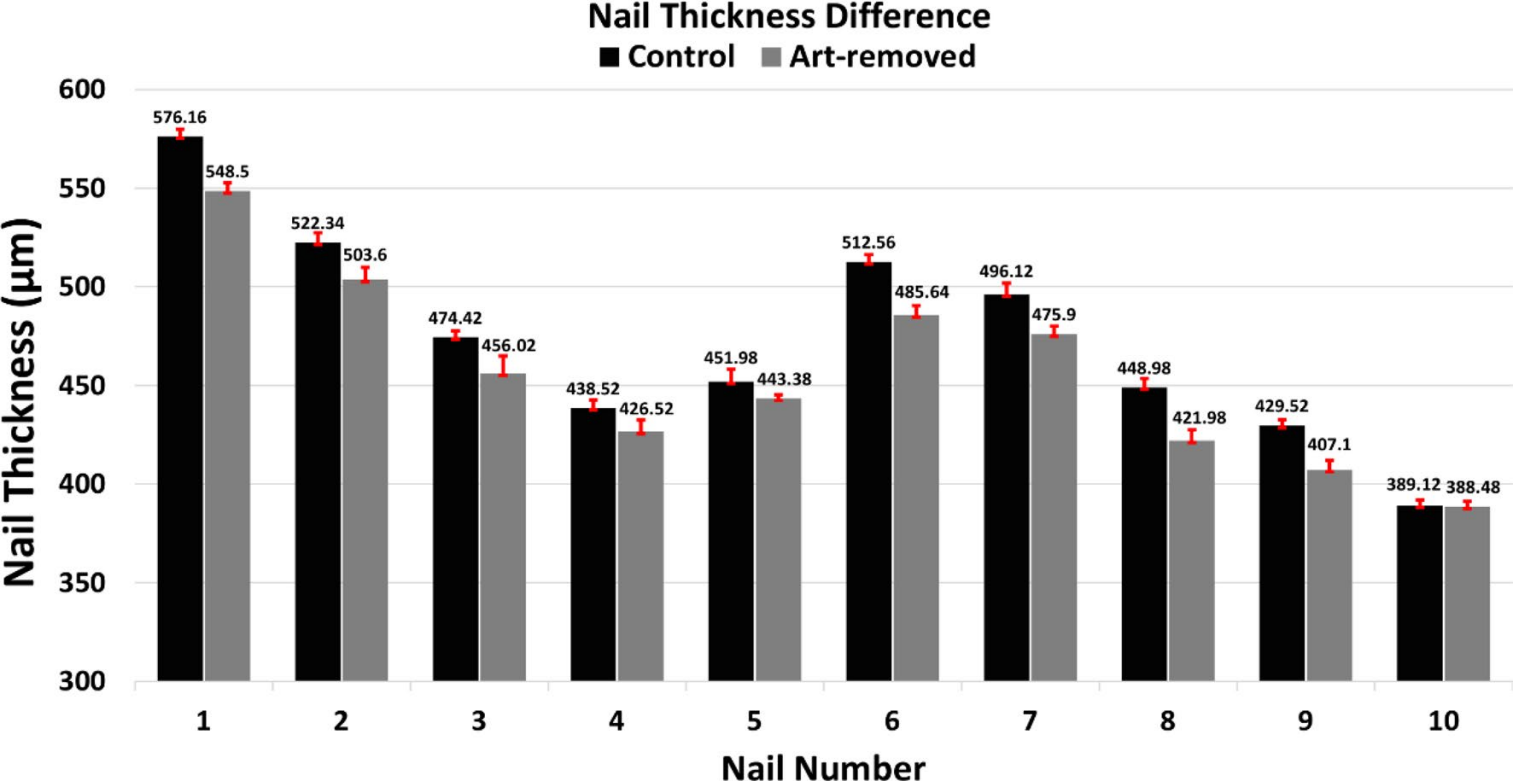
Graphical representation of the thickness difference of control and art-removed nails.

Before the nail-art, the control nails were imaged for five consecutive weeks, and after removing nail-art, art-removed nails were imaged for five consecutive weeks. In Fig. 9b, black, blue, green, magenta, and red color intensity profiles represent five weeks of monitored intensity profiles of a nail specimen. Intensity peaks from the dorsal, intermediate, and ventral sublayers can be detected in the intensity profile, shown in Fig. 9b. The intensity peaks from the top surface of the nail bed can also be detected in the intensity profiles of a nail specimen. To measure the thickness difference between control and the art-removed nails, the intensity peak position of the nail bed top surface in the x-axis was measured from the intensity profile of both control and art-removed nails. Nail thickness measurement data of control and art-removed nails are shown in Table 1. In Table 1, nail thickness and standard deviation values of both control and art-removed nails are the averaged of intensity peak positions of the nail bed top surface measured from intensity profiles of consecutive five weeks of imaging. Figure 10 shows a graphical representation of the thickness difference of control and art-removed nails of right and left hands.

**Table 1:** Thickness difference of control and art-removed nails.

Nail #	Thickness (µm) (control)	Standard deviation (µm) (control)	Thickness (µm) (after nail-art removal)	Standard deviation (µm) (after nail-art removal)	Nail thickness difference (µm)
1	576.16	3.750	548.5	4.253	27.66
2	522.34	5.046	503.6	6.230	18.74
3	474.42	3.143	456.02	8.923	18.4
4	438.52	4.080	426.52	5.929	12
5	451.98	6.158	443.38	1.851	8.6
6	512.56	3.750	485.64	4.858	26.92
7	496.12	5.710	475.9	4.107	20.22
8	448.98	4.572	421.98	5.458	27
9	429.52	3.143	407.1	4.908	22.42
10	389.12	2.651	388.48	2.737	0.64

**Figure 11:**
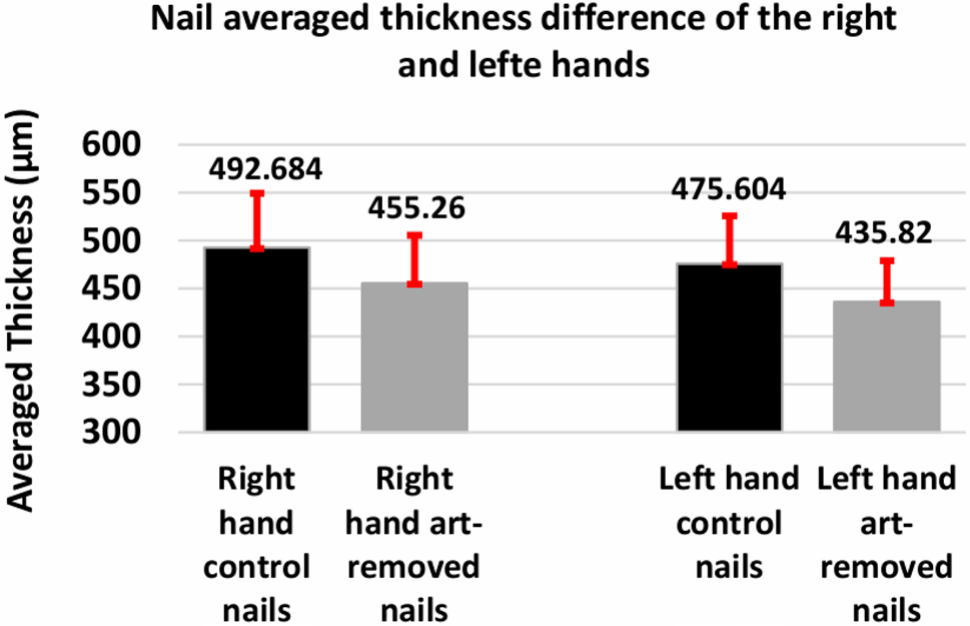
Graphical representation of nail averaged thickness difference of control and art-removed nails of the right and left hands.

The graphical representation of the nail averaged thickness difference of control and art removed nails of the right and left hands is shown in Fig. 11. The right-hand control and art-removed nails averaged thicknesses were measured 492.684 and 455.26 µm, respectively, and the left-hand control and art-removed nails averaged thicknesses were measured 475.604 and 435.82 µm, respectively. The thicknesses of the thumb, index, middle, ring, and little fingernails were averaged for both the right and left hands and shown in Fig. 11. The nail plate thickness increase immediately after the nail bath and it is a short-term effect. After 15 min the nail plate thickness goes back to its initial position^19^. In this study, a nail bath was not applied and the art removed nails were imaged for 5 consecutive weeks after removing the nail-art, and then A-scan profiles were averaged to measure the nail plate thickness, thus, the art-removed nail thickness result was free from the moisture and water content effects.

From an imaging point of view, the whole fingernail in vivo 3D imaging with a large scan area, and without any motion artifact was challenging. Figure 6 shows the 3D images of control, with nail-art, and art removed nail 3D images without any motion artifact, thus, the quantitative assessment of surface quality and micro-crack on the nail plate surface is successfully demonstrated. The inner structure of the nail plate can be analyzed through 2D and 3D enface images (shown in Figs. 4 and 7), which were acquired from motion artifact less 3D imaging. Figure 5B1–B10 shows the shiny, transparent, semi-transparent, and non-transparent properties of different kinds of nail-arts and their inner appearance in nail-art practice. Also, in 15 weeks nail monitoring period, the reference arm power was kept unchanged to detect the backscattered intensity difference of control and art-removed nail, which is shown in Fig. 8. The depth intensity profile of nail 2D-OCT images in this study was normalized by dividing the maximum acquired intensity. The intensity of all nail 2D-OCT images was high in the nail surface area and after the normalization process, the surface intensity was found 1 for all nail images. Thus, the surface intensity of control and art removed nail images shown in the depth intensity profiles in Figs. 3, 8, and 9 is the same, and in the depth direction, the intensity is different because of the varying refractive index and change in the inner biological structure of nail plate over a long imaging/monitoring period. However, the optical imaging systems such as CLSM, OCT, which work based on the back-scattered intensity from the sample, were used for the diagnosis of different nail diseases^20,45^. Back-scattered intensity plays an important role in the diagnosis of different nail diseases and disorders because the back-scattered intensities from the healthy and unhealthy nail plate can be different due to the change of refractive index and the inner biological structure of the nail plate.

In this study, although only one test subject was examined using the OCT system to visualize post-progressive nail-art effects on the nail plate, this study can make a foundation of the nail-art effect assessment in the future, because the monitoring of in vivo fingernails for a long period using OCT system has not been studied yet. Nail plate thining tendency by the effect of various types of nail-art has already been reported by several research groups^3–5^. It is also reported that the nail plate can be thickened for various nail disease^45–47,49^, not by the nail-art. In this study, the nail plate thinning tendency of ten fingernails shows the nail-art practice effects on the nail plates. However, the assessment of back-scattered intensity and nail thickness difference of control and the art-removed nails shown in Figs. 8 and 10, respectively, is a feasibility study and its result can be improved in the future by examining a large number of test subjects.

## Conclusion

The SD-OCT system was applied in this study as a diagnostic tool for detecting nail-art effects on fingernails. Control specimens, with nail-art, and nail-art removed fingernails of a volunteer were imaged for 15 weeks. The monitored results revealed that the nail-art practice caused some cracks and white patches on the surfaces of art-removed nail plates. No nail cracks were detected on the nail plate surface during the nail monitoring period of control and with nail-art. After 72 h of removing nail-art, some cracks were detected in the sub-surface layers of the nail plate surface and the progression of cracks was confirmed after 1 and 2 weeks of nail-art removal. Two dimensional (2D-OCT) images of control and art-removed nails were also analyzed by an in-house signal processing algorithm to determine the change in backscattered intensity and nail thickness. The monitored results also revealed that the backscattered intensity of all control nails was higher than the backscattered intensity of art-removed nails and the thickness of art-removed nails was reduced compared to control specimens. To the best of our knowledge, this is the first study, where the feasibility of OCT imaging modality is assessed in monitoring in vivo fingernails for long period in the diagnosis of nail plate abnormalities and irregularities as the post-progressive nail-art effect on the nail plate. From these results, it can be concluded that the OCT imaging technique can be used effectively as a diagnostic tool to investigate the effects of nail-art and the abnormalities on the nail plate that cannot be detected with the naked eye, which can provide real-time imaging and a large scanning volume with high resolution compared with other conventional imaging methods of fingernail assessments.
